# Management of congenital muscular dystrophies related to defects in the *LMNA* gene

**DOI:** 10.1186/1750-1172-10-S2-O24

**Published:** 2015-11-11

**Authors:** Susana Quijano-Roy, Adele D'Amico

**Affiliations:** 1 AP-HP, Service de Pédiatrie, Pôle Pédiatrique, Hôpital R. Poincaré, Garches. Hôpitaux Universitaires Paris-Ile-de-France Ouest; GNMH, UVSQ, France; 2 Unit of Neuromuscular and Neurodegenerative Disorders, Bambino Gesù Children's Hospital, Rome

## 

Congenital Muscular Dystrophies are a heterogeneous group of muscular disorders defined as early onset muscle weakness and progressive course associated to dystrophic features at muscle biopsy. CMDs related to lamina A/C gene (*LMNA*) defect include different phenotypes that can be classified as *i)* severe phenotype with generalized muscular weakness and contractures by birth, *ii)* ‘dropped head’ phenotype with prominent involvement of axial muscles that generally evolves to rigid spine phenotype and *iii)* early Emery-Dreifuss phenotype. All these condition generally lead in the first 2 decades to cardiac disturbances, respiratory insufficiency, orthopedic complication and metabolic disorders. The clinical management requires a multidisciplinary and rigorous approach focused on early medical and rehabilitative interventions with the main aims to prevent ‘fatal heart event’, to cure co-morbidities (pulmonary insufficiency and spinal and joint contractures) and to improve the quality of life of these children.

